# Response dataset from canine extramedullary plasmacytoma treated with lipophobic drugs enhanced by electroporation

**DOI:** 10.1016/j.dib.2020.106085

**Published:** 2020-07-29

**Authors:** Denner Santos dos Anjos, Ygor Amaral Rossi, Oscar Rodrigo Sierra, Cynthia Marchiori Bueno, Andrigo Barboza De Nardi, Carlos Eduardo Fonseca-Alves

**Affiliations:** a Department of Veterinary Clinic and Surgery, São Paulo State University (UNESP), Jaboticabal, São Paulo, Brazil; b Eletro-Onkovet Service; c Veterinary Student, University Franca (UNIFRAN), Franca, São Paulo, Brazil; d Department of Veterinary Surgery and Animal Reproduction, School of Veterinary Medicine and Animal Science, São Paulo State University (UNESP), Botucatu, São Paulo, Brazil; e Institute of Health Sciences, Universidade Paulista – UNIP, Bauru, São Paulo, Brazil

**Keywords:** Bleomycin, Cisplatin, Electropermeabilization, Electric stimulation therapy, Neoplasms, Plasma cell tumor

## Abstract

Over the past 15 years, lipophobic drugs, such as bleomycin and cisplatin, have been used combined with electroporation, which promotes their uptake through the cell membrane. The present data describe general findings following electrochemotherapy and how plasmacytomas can respond to this technique. We will explain and illustrate specific outcomes during the remission process. The data presented here can be useful for researchers, veterinarians, and pet owners. Furthermore, the data could be useful for other cutaneous or oral tumors in which electrochemotherapy may be indicated. Interpretation of the data and outcomes may be found in the research article entitled “Outcome following curative-intent electrochemotherapy for extramedullary plasmocytoma in dogs – case reports .”

**Specifications table**

**Table:** 

**Subject**	Veterinary Science and Veterinary Medicine (General)
**Specific subject area**	Veterinary oncology, bioelectrochemistry
**Type of data**	Table Figure Text
**How data were acquired**	Photographs were taken before the session to follow-up outcomes
**Data format**	Raw and analyzed data
**Parameters for data collection**	All patients were planned to undergo electrochemotherapy exclusively. Prior to the session, tumor volume was measured using a caliper for tumor volume and photographs were taken to analyze local adverse effects post-treatment. All patients had good health status on physical exam.
**Description of data collection**	All patients were followed weekly for 4 weeks post electrochemotherapy (ECT) treatment to evaluate tumor response after the initial approach. In addition, patients were followed-up monthly to evaluate recurrence post-ECT.
**Data source location**	Cases were obtained from a private veterinary clinic (CEMA-Batatais) and specialized electrochemotherapy service (Eletro-Onkovet- Franca). City: Batatais and Franca Region: São Paulo Country: Brazil
**Data accessibility**	With the article

**Value of the data**

Electroporation combined with lipophobic drugs could be an effective approach for cutaneous and oral tumors when the anatomic region is not amenable for surgery.
Electrochemotherapy for plasma cell tumors induced remission in these cases and could be indicated as the sole therapy.
Data set involves the outcomes and local adverse effects in canine cases after electrochemotherapy, reaffirming that veterinarians and owners should know this evolution to provide appropriate care.

## 1 Data description

This data set contain information regarding the use of lipophobic drugs, such as bleomycin and cisplatin, combined with electric pulses (electroporation) to increase their uptake through the aqueous pathway (nanopores) by the cell membrane. Additionally, this data set contains five figures and one table that depict the mechanism of action and outcome of tumors after electrochemotherapy. All patients underwent tumoral staging, including abdominal ultrasonography and thoracic radiography. The complete blood count and biochemistry profile were also performed. No abnormalities were observed. Peripheral or abdominal lymph node involvement was not observed.

Local adverse effects, such as ulceration and edema that healed within 7–21 days, were observed in all dogs. Due to adverse effects (i.e., edema and ulceration), patients usually received non-steroidal anti-inflammatory drugs, such as meloxicam (at a dose of 0.1 mg/kg once daily for seven days), and antibiotics, such as enrofloxacin (at a dose of 5 mg/kg twice daily for 5 days), after ECT. Some patients may require extended supportive care after ECT based on macroscopic healing of the wound (Denner Santos Dos Anjos, personal observation).

According to the criteria for dermatologic/cutaneous adverse events following biological antineoplastic therapy in dogs and cats (VCOG-CTCAE v1.1) [1], one dog developed grade I (combined area of ulcers <1 cm^2^, non-blanchable erythema of intact skin with associated warmth or edema) and three dogs developed grade II (combined area of ulcers 1–2 cm^2^, partial thickness skin loss involving the skin or subcutaneous fat) lesions.

**Fig. 1:**
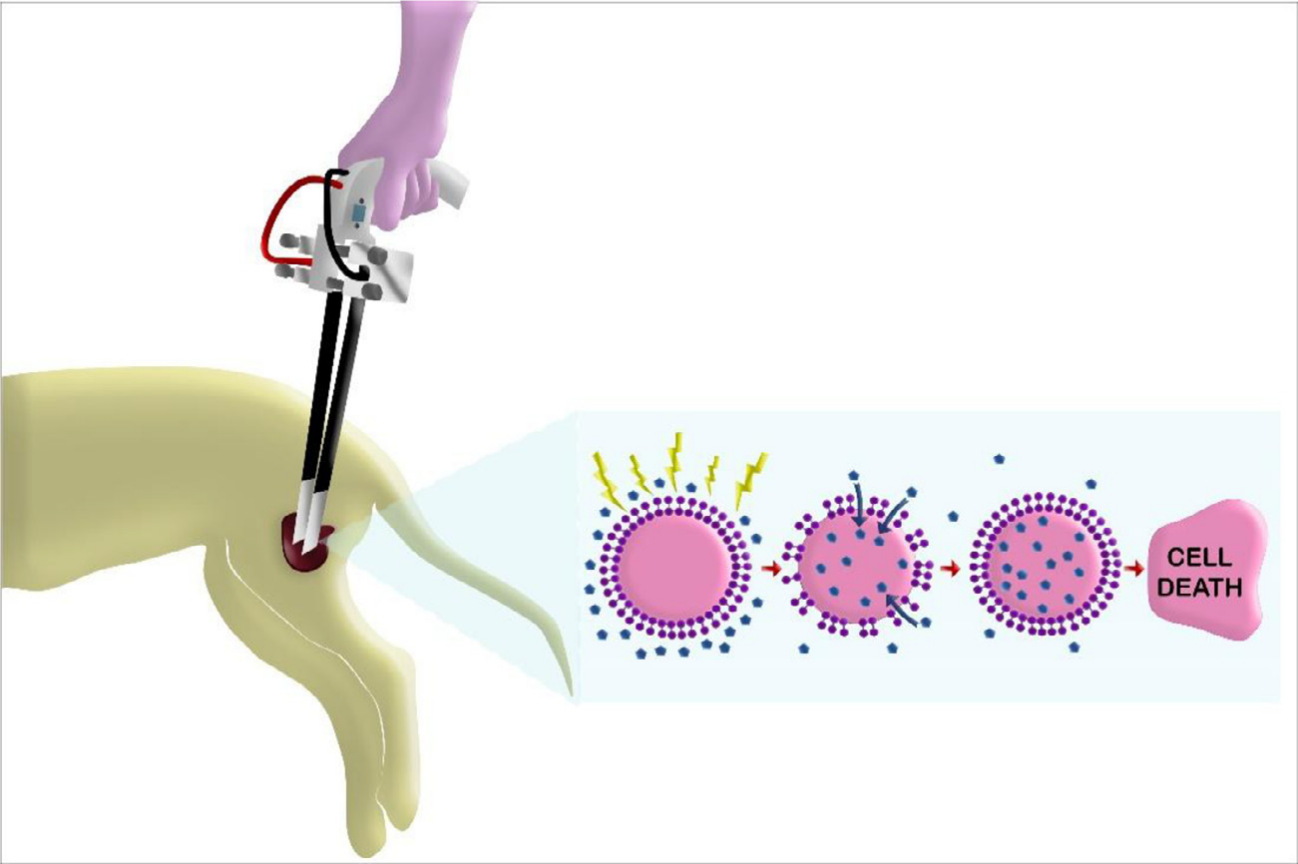
Illustrative schematic of one of the mechanisms of electrochemotherapy (from left to right side). First, the plate electrode covers a part of the cutaneous tumor. Once the electric field is delivered over the tumor by the electroporator (Onkodisruptor®), the electric pulses lead to the creation of nanopores in the cell membrane promoting a movement of large amount of drug (specially lipophobic drugs) into the cell. Resealing of the membrane then occurs once the electric field has stopped, leading to cell death.

Fig. 1 shows the use of an electrode plate (Onkodisruptor® device) on a cutaneous tumor. During the pulse, an electric field is generated over the cell membrane that creates nanopores; after the pulse has stopped, the membrane is resealed. It is possible to notice the increased uptake (blue spots) of antineoplastic drug by the pores, promoting higher intracytoplasmic drug concentrations, leading to cell death.

One of the mechanisms of ECT is increased cell membrane permeability following the delivery of permeabilizing electrical pulses. This temporary permeabilization allows free transit of molecules, such as bleomycin or cisplatin, ions, and water, between the two sides of the cell membrane [2]. In addition, ECT also results in disruption of the tumor vasculature without affecting the adjacent normal vasculature. Moreover, the application of electric pulses also induces vasoconstriction of blood vessels (vascular lock), endothelial apoptosis, and temporary hypoxia [3]. Finally, ECT boosts the immune system by increasing the infiltration of dendritic cells, which stimulate immunogenic cell death and decrease regulatory T-cell levels, as observed in melanoma patients [4,5]

**Fig. 2:**
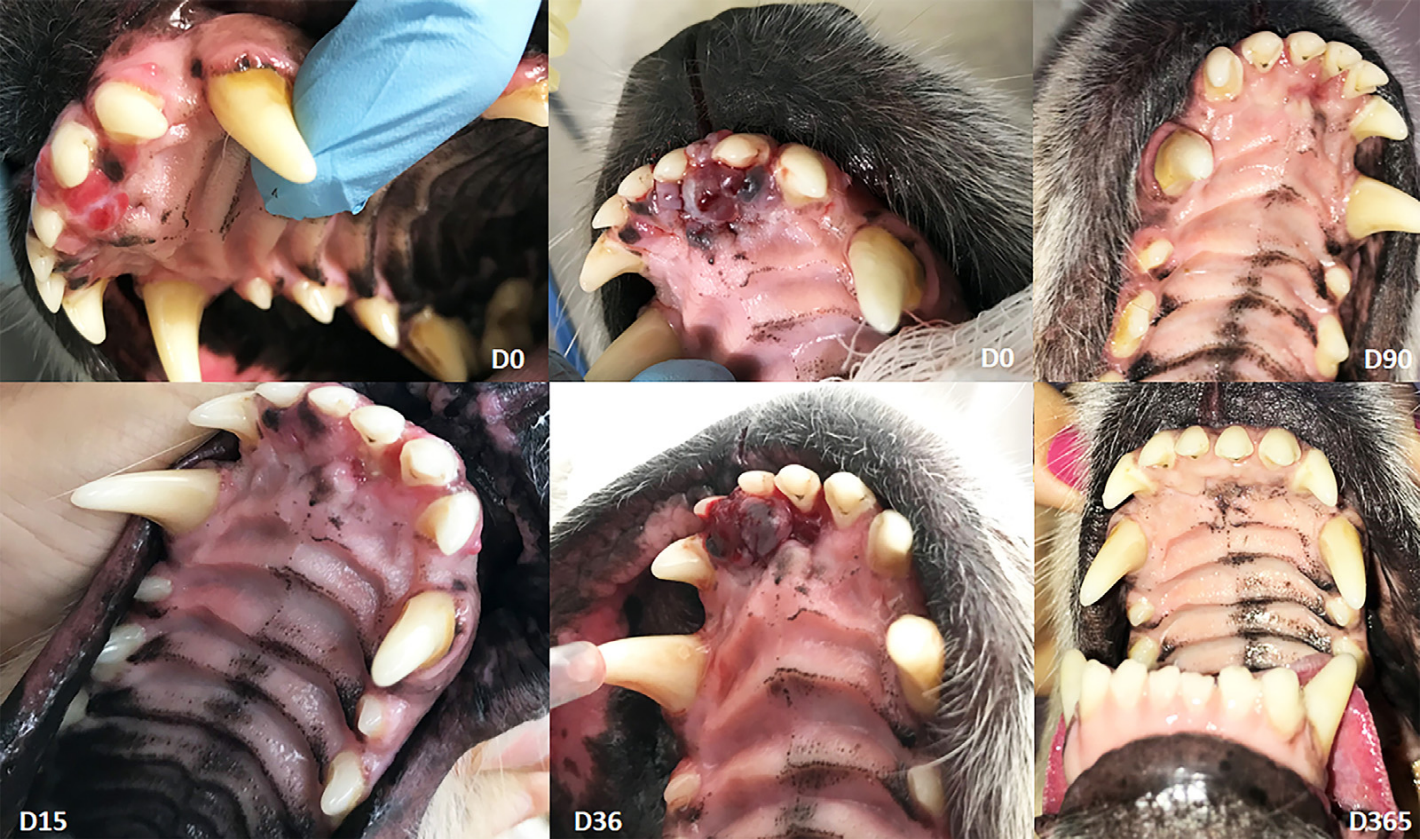

**Fig. 3:**
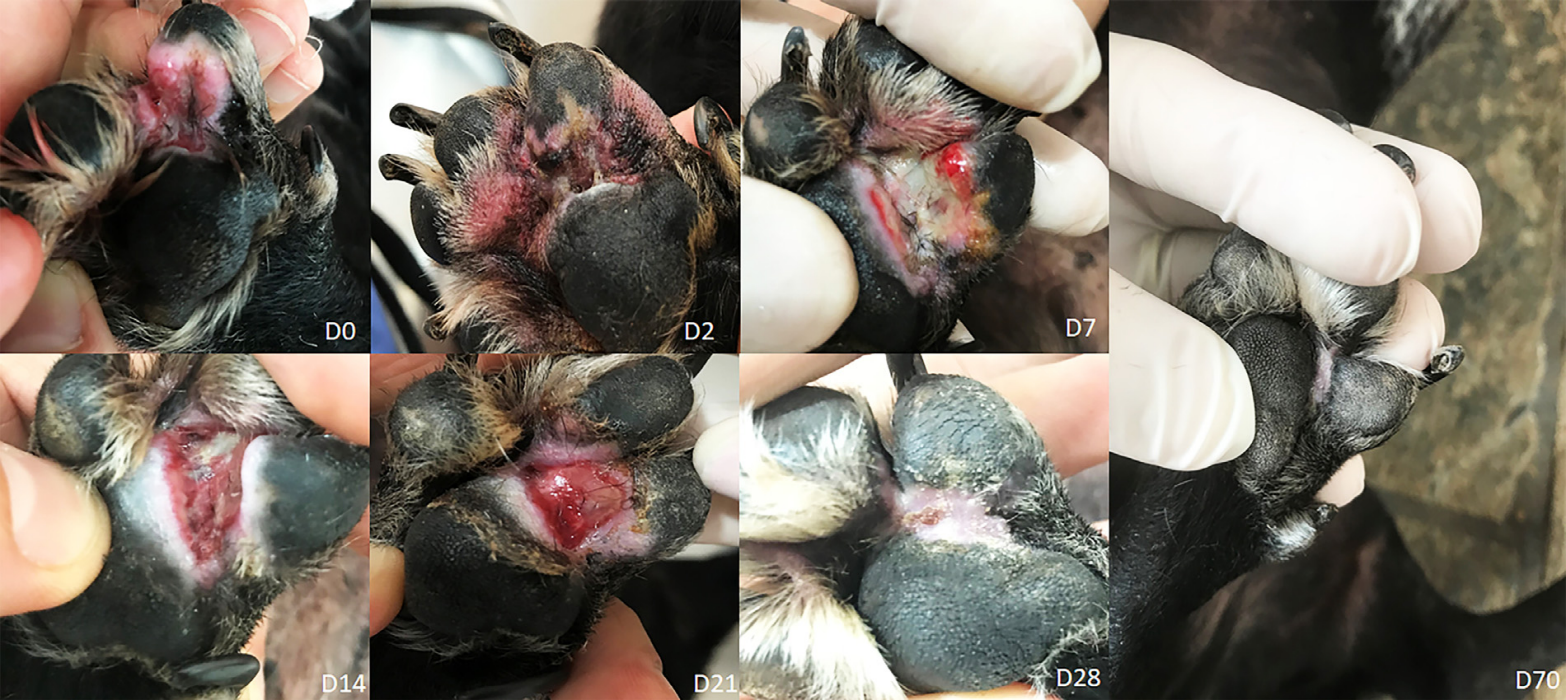

Fig. 2 demonstrates an oral canine case in the maxillary incisor region. It is possible to notice the immediate vascular lock (D0) after electric pulses suggestive of vasoconstriction and hypoxia, as described in literature [2,3]. Fig. 3 shows a digital plasma cell tumor with crust formation after two days of ECT (D2) evolving to ulceration and erythema by day 7 (D7), with complete healing by day 28 (D28).

**Fig. 4:**
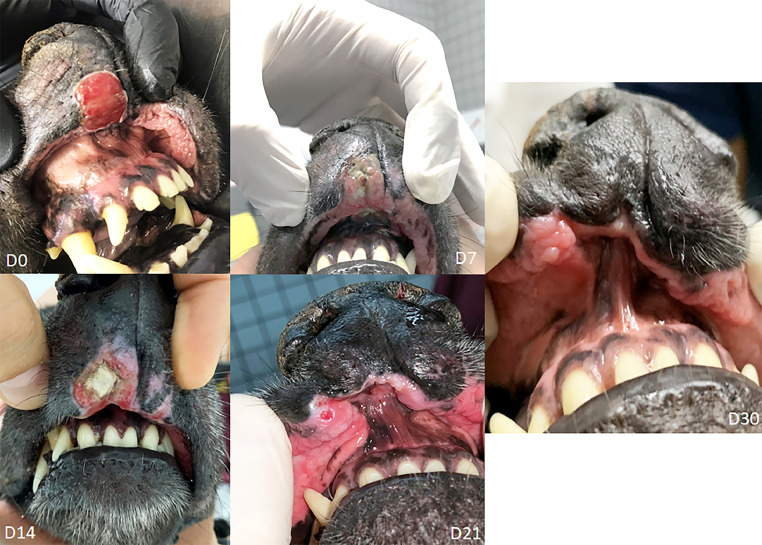

**Fig. 5:**
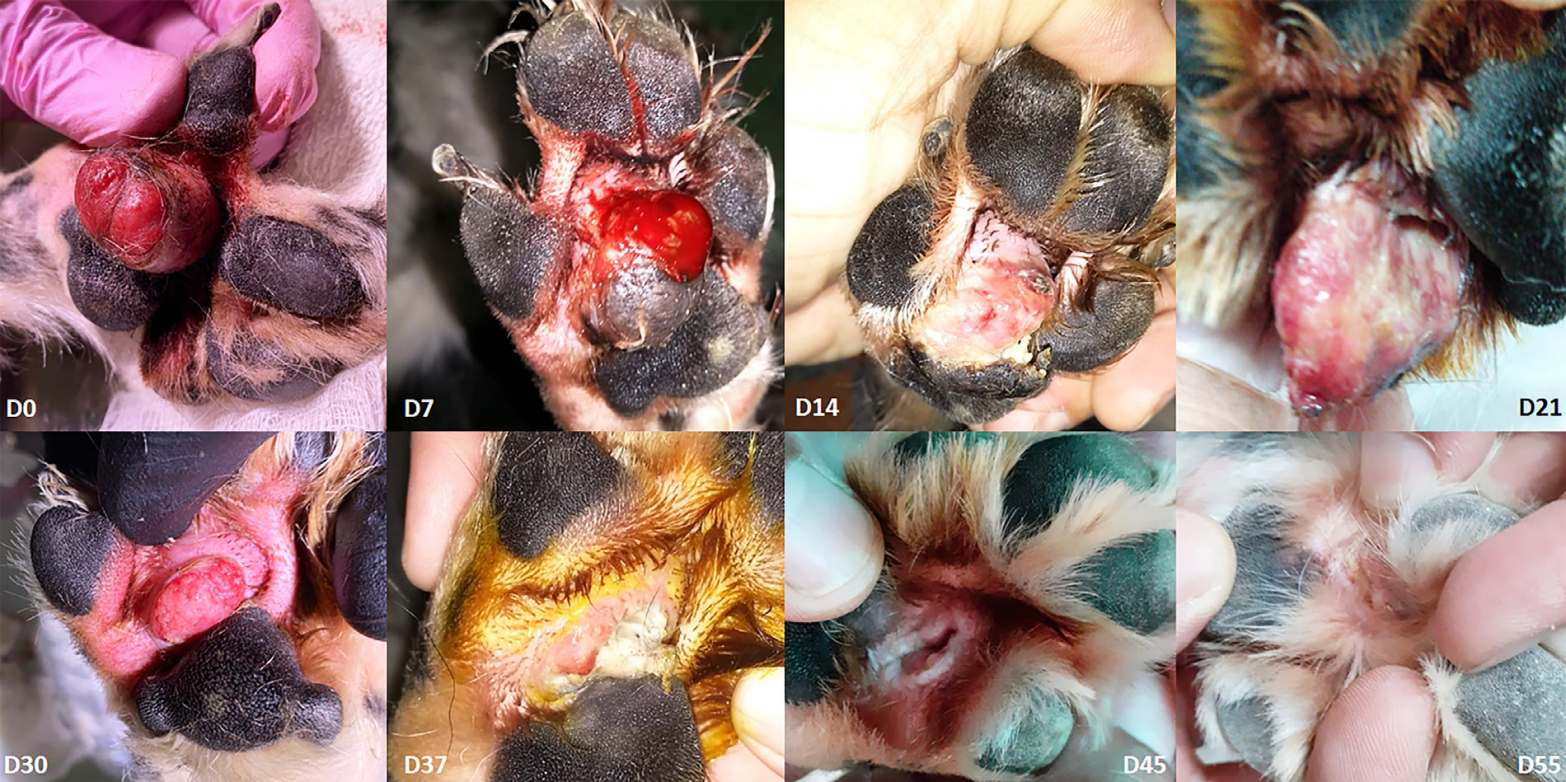

Fig. 4 shows the formation of an ulcer in the upper lip post-ECT in a process of healing lasting 21 days. Fig. 5 demonstrates a cutaneous tumorous lesion in the right pelvic limb between the pads. Although vascular lock caused by electroporation is expected during the remission process, this patient presented on day 7 post-ECT (D7) with a hemorrhagic lesion, possibly due to mechanical trauma. Cold therapy using ice was indicated locally for 10 minutes twice a day in order to minimize bleeding. On day 14 (D14), an ulcer developed and on day 21 (D21), a fistula had developed. A second ECT session on day 30 was necessary to achieve complete remission.

Table 1 summarizes clinical information, electrical parameters, response to electrochemotherapy, and disease-free interval in each case.

**Table 1:** Summarizes the clinical information, containing electric parameters and results obtained in canine extramedullary plasmocytoma submitted by ECT.

ID	Breed	Age (years)	Location	Tumor volume (V=axb²π/6)	Electroporator	Pulse Protocol	Drug (1st session)	Drug (2nd session)	Respose to treatment (at 4w)	Response duration (after 4w)	Response duration (Disease-Free-Interval) days
1	Golden-Retriever	8	Maxillary incisive	0.1 cm³	LC-BK100¹	One train 8 square pulses 1000V/cm	Cisplatin	Bleomycin*	RC	8 days	>515
2	Mixed	10	Interdigit (right pelvic limb)	0.13 cm³	LC-BK100¹	One train 8 square pulses 1000V/cm	Bleomycin	No	RC	>695	>695
3	Mixed	14	Upper lip	1.1 cm³	Onkodisruptor^2^	Five trains 8 biphasic pulses 1000V/cm	Cisplatin	No	RC	> 240	> 240
4	Border Collie	6	Paw (right pelvic limb)	5.3 cm³	Onkodisruptor^2^	Five trains 8 biphasic pulses 800 V/cm	Cisplatin	Cisplatin*	PR (1.33 cm³)	-	> 90

¹In-house clinical electroporator (São Paulo, Brazil)

²Certificated electroporator (Biopulse, S.r.l., Naples, Italy)

⁎ ID 1 received a second session at day 36th due to new lesion; ID 4 received a second session at day 30th due to stable disease after first ECT session. RC: complete remission; PR: partial remission; pi: 3.14

## 2 Experimental design, materials, and methods

The experimental design, materials, and methods were based on a study by Dos Anjos et al. [6]. Four client-owned canine patients with extramedullary plasmacytoma were enrolled in this retrospective study. The patients in this study underwent ECT exclusively due to the anatomic region where the lesion was located, since radical surgery was declined by the owners. Table 1 describes the electric parameters for each case and their results.

Before ECT treatment, the patient was subjected to trichotomy (in cases of cutaneous tumors), shaving around the lesions so the plate electrodes made better contact. In this way, a good contact between the plate electrodes and the skin was assured by depilation and application of a conductive gel to the treatment area. Two electrical pulse protocols were used (Table 1):

Protocol 1: After intratumoral cisplatin administration or five minutes after intravenous bleomycin (rescue protocol in case 1 and first choice in case 2), eight square pulses at 1,000 V/cm (standard parameter), at 1 Hz frequency, lasting 100 µs each were administered using an in-house fabricated clinical electroporator (LC BK-100, São Paulo, Brazil) with six needle electrodes arranged in rows (parallel array) (case 1 and 2) to achieve complete coverage of the lesion.

Protocol 2: After intratumoral cisplatin administration five trains of eight biphasic pulses at 800–1,000 V/cm, at 1 Hz frequency, lasting 50 + 50 µs with a 300 µs interpulse period (total treatment time per train 3.2 ms) were administered using a clinical electroporator for veterinary application (Onkodisruptor®, Biopulse S.r.l., Naples, Italy) (case 3 and 4) with a plate or single-pair needle array electrode to achieve complete coverage of the lesion.

The decision for a second session of ECT in patients 1 and 4 was based on the response evaluation criteria in solid tumors (RECIST) and drug availability. As patient 1 achieved a complete remission by day 30, we followed-up the patient; however, a new lesion was observed on day 36 which led to a second ECT session with a rescue protocol using bleomycin (Table 1). In patient 4, we observed partial remission and stable disease on day 30, so we performed a second ECT session to achieve complete remission. In addition, in some countries, bleomycin is not available on the market and its use has been limited in some patients (especially in large breeds due to their size) because of the larger amount of bleomycin used. In this way, the reasons for their use in each case is explained:

- CASE 1 received cisplatin as first choice, but complete remission only lasted for a short period. Rescue protocol with bleomycin was used during recurrence.
- CASE 2 received bleomycin as first choice (small mixed breed).
- CASE 3 received cisplatin as first choice because of the lack of bleomycin.
- CASE 4 received cisplatin as first choice because of the lack of bleomycin.

In order to determine the amount of drug used in each case, the tumor volume was estimated using the formula V=ab²π/6 (where a is the larger diameter of the tumor nodule and b is the diameter of the tumor nodule perpendicular to a). Additionally, the RECIST system for measurement and assessment of response of tumor lesions was used at 4 weeks post-treatment [7,8].

## Ethics statement

This study was performed in accordance with the National and International Recommendations for the Care and Use of Animals (National Research Council). Informed written consent of owners was obtained prior to treatment for all dogs.

## Declaration of Competing Interest

The authors declare that they have no known competing financial interests or personal relationships which have, or could be perceived to have, influenced the work reported in this article.

## Appendix Supplementary materials

Image, application 1
